# Safety of catheter ablation for atrial fibrillation during hospitalization for acute heart failure: a nationwide analysis from the jROAD-dpc database

**DOI:** 10.1093/europace/euag273

**Published:** 2026-09-23

**Authors:** Haruka Matsuura, Koshiro Kanaoka, Shoko Chishaki, Koki Takegawa, Yoshitaka Iwanaga, Tetsuhisa Kitamura, Yoshihiro Miyamoto, Kengo Kusano

**Affiliations:** Department of Medical and Health Information Management, National Cerebral and Cardiovascular Center, 6-1 Kishibe-Shimmachi, Suita, Osaka 564-8565, Japan; Department of Advanced Cardiovascular Medicine, Graduate School of Medical Sciences, Kumamoto University, Kumamoto, Japan; Department of Medical and Health Information Management, National Cerebral and Cardiovascular Center, 6-1 Kishibe-Shimmachi, Suita, Osaka 564-8565, Japan; Department of Medical and Health Information Management, National Cerebral and Cardiovascular Center, 6-1 Kishibe-Shimmachi, Suita, Osaka 564-8565, Japan; Department of Medical and Health Information Management, National Cerebral and Cardiovascular Center, 6-1 Kishibe-Shimmachi, Suita, Osaka 564-8565, Japan; Department of Medical and Health Information Management, National Cerebral and Cardiovascular Center, 6-1 Kishibe-Shimmachi, Suita, Osaka 564-8565, Japan; Department of Cardiology, Sakurabashi-Watanabe Hospital, Osaka, Japan; Department of Medical and Health Information Management, National Cerebral and Cardiovascular Center, 6-1 Kishibe-Shimmachi, Suita, Osaka 564-8565, Japan; Department of Medical and Health Information Management, National Cerebral and Cardiovascular Center, 6-1 Kishibe-Shimmachi, Suita, Osaka 564-8565, Japan; Department of Cardiovascular Medicine, National Cerebral and Cardiovascular Center, Osaka, Japan

**Keywords:** Atrial fibrillation, Acute heart failure, Catheter ablation, Ischaemic stroke, JROAD-DPC

## Introduction

Catheter ablation is an established rhythm-control strategy for patients with atrial fibrillation (AF) and heart failure (HF), improving left ventricular function and reducing HF hospitalization and mortality.^1,2^ Recent studies suggested potential benefits of early rhythm control or catheter ablation after HF hospitalization, and urgent AF ablation is increasingly being used in clinical practice.^3,4^ However, the safety of AF ablation during hospitalization for acute heart failure (AHF) remains uncertain. AHF is a prothrombotic and haemodynamically vulnerable state that may increase periprocedural thromboembolic risk. Stroke during or after catheter ablation is regarded as one of the most serious procedure-related complications,^5^ highlighting the importance of assessing periprocedural stroke risk. We therefore evaluated the periprocedural safety of AF catheter ablation during AHF hospitalization using a nationwide Japanese database.

## Methods

This retrospective cohort study used the Japanese Registry of All Cardiac and Vascular Diseases–Diagnosis Procedure Combination (JROAD-DPC) database, a nationwide claims-based database covering more than 1,000 hospitals across Japan. The National Cerebral and Cardiovascular Center Ethics Committee approved the study (R25008), and informed consent was waived because anonymized data were used.

We identified adults with HF who underwent first-time AF catheter ablation between April 2012 and March 2024 Patients emergently admitted with worsening HF who underwent ablation during the same hospitalization were classified as the AHF group; those electively admitted for AF ablation with chronic HF were classified as the elective CHF group. The primary analysis excluded patients with severe preprocedural instability, defined as intensive or high-care unit treatment the day before ablation, mechanical circulatory support, mechanical ventilation, or continuous oxygen therapy.

The primary outcomes were thromboembolic events, major bleeding, and in-hospital mortality. Procedure-related complications were defined as events occurring after ablation and before discharge. Thromboembolic events included ischaemic stroke and systemic embolism. Ischaemic stroke was defined as cerebral infarction accompanied by a stroke-specific procedure, medication, or rehabilitation. Systemic embolism was defined as acute arterial occlusion outside the cerebral circulation, excluding pulmonary embolism; major bleeding was intracranial haemorrhage or bleeding requiring a haemostatic procedure or transfusion. Propensity scores incorporated demographics, comorbidities, frailty score, AF type, ablation technology, and hospital characteristics. Adjustment used propensity score matching and stabilized inverse probability of treatment weighting (IPTW), with covariate balance assessed using standardized mean differences (SMDs).

## Results

The primary cohort included 3,675 patients in the stabilized AHF group and 47,960 in the elective CHF group. The proportion undergoing AF ablation during AHF hospitalization increased from 0.5% in 2013 to 1.2% in 2023. Before adjustment, patients in the stabilized AHF group were older, more often female, and had higher CHADS_2_ scores, more comorbidities, and lower functional status (*Figure 1A*). Time from admission to ablation (15.7 ± 10.2 vs. 2.5 ± 2.3 days) and total hospital stay (24.3 ± 15.6 vs. 6.0 ± 5.6 days) were longer in the stabilized AHF group.

**Figure 1:**
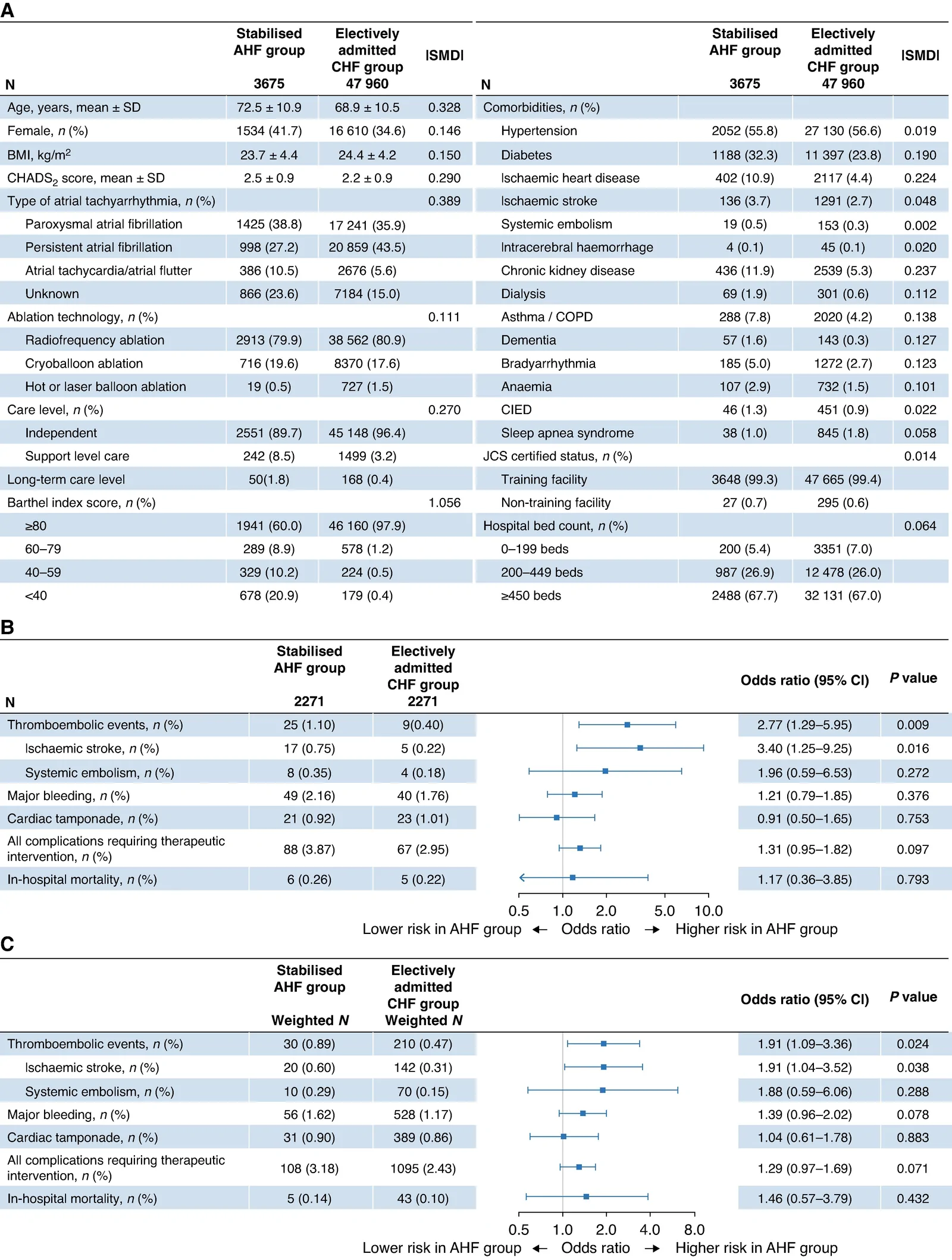
Baseline characteristics and clinical outcomes after adjustment. (*A*) Baseline characteristics of patients in the stabilized acute heart failure (AHF) group and the electively admitted chronic heart failure (CHF) group before adjustment. (*B*) Clinical outcomes after propensity score matching. Odds ratios (ORs) with 95% confidence intervals (CIs) are shown for the stabilized AHF group compared with the elective CHF group. (*C*) Clinical outcomes after stabilized inverse probability of treatment weighting. Weighted event numbers and percentages are shown for each group, together with ORs and 95% CIs. AHF, acute heart failure; BMI, body mass index; CHADS2, congestive heart failure, hypertension, age ≥75 years, diabetes mellitus, and prior stroke or transient ischaemic attack; CHF, chronic heart failure; CI, confidence interval; CIED, cardiac implantable electronic device; COPD, chronic obstructive pulmonary disease; IPTW, inverse probability of treatment weighting; JCS, Japanese Circulation Society; OR, odds ratio; PSM, propensity score matching; SD, standard deviation; SMD, standardized mean difference.

After propensity score matching, 2,271 well-balanced pairs were analysed, with all baseline covariates, including anticoagulant use, achieving SMDs <0.1. Thromboembolic events were more frequent in the stabilized AHF group (1.10% vs. 0.40%; odds ratio [OR] 2.77, 95% confidence interval [CI] 1.29–5.95; P = 0.009). This was mainly driven by ischaemic stroke (0.75% vs. 0.22%; OR 3.40, 95% CI 1.25–9.25; P = 0.016), with no significant difference in systemic embolism (*Figure 1B*). Major bleeding, cardiac tamponade, complications requiring therapeutic intervention, and in-hospital mortality did not differ significantly between groups. IPTW analysis showed similar findings, with higher rates of thromboembolic events and ischaemic stroke in the stabilized AHF group, but no significant differences in other outcomes (*Figure 1C*). In sensitivity analyses including patients with severe preprocedural clinical status, major bleeding and in-hospital mortality were also more frequent in the AHF group.

## Discussion

Major periprocedural complications after AF catheter ablation are rare, making large-scale real-world data essential for evaluating procedural safety. In this nationwide cohort study using the JROAD-DPC database, AF catheter ablation during hospitalization for AHF was associated with a higher risk of periprocedural thromboembolic events than elective catheter ablation in patients with CHF. This excess risk was mainly driven by ischaemic stroke and remained consistent across sensitivity analyses. In contrast, major bleeding, in-hospital mortality, and procedure-related complications did not differ between groups. The incidence of periprocedural complications was broadly consistent with previous registry reports, supporting the validity of our code-based identification of AF catheter ablation and related complications.^6^

Our findings may reflect the prothrombotic milieu of AHF, characterized by blood stasis, endothelial dysfunction, and hypercoagulability. Thromboembolic risk may be further increased during the acute phase by systemic inflammation, neurohormonal activation, haemodynamic instability, and reduced physical activity.^7^ Previous studies have shown that ischaemic stroke risk is approximately 2.8- to five-fold higher within 30 days after HF diagnosis and clusters early during hospitalization for acute decompensated HF.^8,9^ Our findings are consistent with these reports and suggest that AHF hospitalization may represent a vulnerable period for thromboembolic complications, even after apparent clinical stabilization.

Although these findings highlight the need to consider periprocedural thromboembolic risk during hospitalization for AHF, decisions regarding the timing of AF ablation should also take into account its potential clinical benefits. AF ablation is an established treatment associated with improved prognosis in selected patients with heart failure and has been shown to improve long-term clinical outcomes.^1^ In particular, early AF ablation may be clinically important in patients with tachycardia-induced cardiomyopathy or in those with heart failure accompanied by drug-refractory rapid AF.^10^ AF ablation timing in acute heart failure should be individualized according to heart failure status and clinical background, balancing periprocedural thromboembolic risk against the potential benefits of early rhythm control and long-term AF ablation.

This study has some limitations. JROAD-DPC is an administrative database and lacks detailed laboratory and imaging data, information on anticoagulant therapy duration before ablation and adherence, and intraprocedural data. Residual confounding cannot be excluded despite statistical adjustment. Only in-hospital outcomes were captured.

In conclusion, AF catheter ablation during hospitalization for AHF was associated with increased thromboembolic risk, highlighting the importance of careful patient selection and individualized procedural timing.

## Data Availability

The JROAD-DPC data used in this study are owned by the Japanese Circulation Society (JCS) and were provided for research purposes. These data are not publicly available and may not be shared without permission from the JCS.
